# What really impacts the use of active learning in undergraduate STEM education? Results from a national survey of chemistry, mathematics, and physics instructors

**DOI:** 10.1371/journal.pone.0247544

**Published:** 2021-02-25

**Authors:** Naneh Apkarian, Charles Henderson, Marilyne Stains, Jeffrey Raker, Estrella Johnson, Melissa Dancy

**Affiliations:** 1 School of Mathematical and Statistical Sciences, Arizona State University, Tempe, Arizona, United States of America; 2 Mallinson Institute for Science Education, Western Michigan University, Kalamazoo, Michigan, United States of America; 3 Department of Chemistry, University of Virginia, Charlottesville, Virginia, United States of America; 4 Department of Chemistry, University of South Florida, Tampa, Florida, United States of America; 5 Department of Mathematics, Virginia Polytechnic Institute and State University, Blacksburg, Virginia, United States of America; 6 Center for STEM Learning, University of Colorado Boulder, Boulder, Colorado, United States of America; Universidade de Mogi das Cruzes, BRAZIL

## Abstract

Six common beliefs about the usage of active learning in introductory STEM courses are investigated using survey data from 3769 instructors. Three beliefs focus on contextual factors: class size, classroom setup, and teaching evaluations; three focus on individual factors: security of employment, research activity, and prior exposure. The analysis indicates that instructors in all situations can and do employ active learning in their courses. However, with the exception of security of employment, trends in the data are consistent with beliefs about the impact of these factors on usage of active learning. We discuss implications of these results for institutional and departmental policies to facilitate the use of active learning.

## Introduction

Undergraduate science, technology, engineering, and mathematics (STEM) instruction in the United States has faced increasing scrutiny for years, with particular focus on introductory courses. General Chemistry, Introductory Quantitative Physics, and Single-Variable Calculus are three examples of gatekeeper introductory courses—they are high enrollment, high-risk, foundational courses that have outsized impact on students’ pathways to a STEM major [1]. Low passing rates in these courses have drawn much attention, but there is evidence to suggest that negative learning experiences dominate students’ reasons for leaving [2,3]. The percentage of STEM-intending students who complete an undergraduate STEM degree has stayed at roughly 40% since 1997, despite an increasing demand for scientists and technicians with a bachelor’s degree [2,3]. While increasing numbers of women and students of color enter STEM majors, they continue to leave at high rates, indicating a continued and substantial loss of natural talent and interest in the sciences [3]. These persistent concerns have drawn attention from university administrators, researchers, and governing bodies seeking to increase the pool of STEM graduates for economic and social reasons [4].

Decades of research in mathematics and science education has led to the development of active learning instructional strategies that are empirically demonstrated to promote content understanding, attitudes, and retention among all students, and to reduce achievement gaps between dominant and underrepresented groups in STEM [3,5,6]. In the broadest sense, *active learning* refers to classroom strategies that move away from a transmission or “telling” model (the classic “didactic lecture”) toward a model where students actively engage in problem-solving and knowledge creation [5,6]. Specific strategies might leverage individual investigations, team-based problem-solving, and/or whole class discussions. A meta-analysis of 225 studies involving all types of active learning strategies in multiple disciplines found that active learning strategies consistently produce better student learning and reduced failure rates than lecture-based methods [5]. Even when broadly conceived, the use of such strategies in undergraduate STEM courses remains sparse despite concerns about student success rates, and despite increasing awareness of the need to implement active learning [3,7,8]. This paper examines empirical data related to six common beliefs about factors that impact instructors’ use of active learning [9,10]. Three of these beliefs are primarily about contextual factors:

1. Large class sizes hinder the use of active learning.
2. Traditional fixed-seat classrooms hinder the use of active learning.
3. Emphasizing student evaluations of teaching hinders the use of active learning.
  Three are primarily individual instructor characteristics:
4. Not having security of employment (e.g., tenure) hinders the use of active learning.
5. High levels of research activity hinders the use of active learning.
6. Experience with active learning as a student, or as a student instructor, facilitates the use of active learning.

We selected these beliefs, in part, because each can be linked to policy decisions and institutional priority setting. For example, policy decisions related to the contextual factors include: directing institutional funds toward decreasing class sizes by hiring additional faculty; building new classrooms designed to facilitate group work as opposed to new auditorium-style lecture halls; or revising assessments of teaching and their use in professional review. Beliefs about individual characteristics can shape policy decisions by, for example, impacting who is targeted by a change initiative, perhaps focusing on newer faculty and instructors or creating factions of teaching-focused and research-focused faculty. Despite their potential impact on important decisions and priority-setting, strong empirical data related to these beliefs has not previously been available.

## Methods

Data for this report comes from a national survey of postsecondary instructors teaching introductory STEM courses at two-year colleges (TYC), four-year colleges (predominantly undergraduate institutions, PUI), and universities (UNI) in the United States. Data collection was conducted in Spring 2019, and the final sample reported on here consists of 3,769 respondents who were primary instructors of a general chemistry, single-variable calculus, or introductory quantitative physics course in the 2017–18 or 2018–19 academic year. This project was approved under the exempt category of review by the Western Michigan University Human Subjects Institutional Review Board, HSIRB Project Number 17-06-10. Written consent was obtained from survey participants.

### Data collection

A new survey instrument was developed by the six authors for this project. The full survey covered five main topics: (1) course context and details; (2) instructional practice; (3) awareness and usage of active learning instruction; (4) perceptions, beliefs, and attitudes related to students, learning, and departmental context; (5) personal demographics and experience. Specific items and the overall format were informed by previous large studies of chemistry [8,11,12], mathematics [13,14], and physics education [15,16]. The exact wording of the survey items included in this analysis are included in the supplement. When possible, previously validated instruments and scales were reproduced in their entirety as part of the survey. A web-based version of the instrument was built and distributed in partnership with the American Institute of Physics Statistical Research Center. Stratified random sampling was done by institution based on institution type, with the goal of developing a representative sample of institution types. All potential participants at each selected institution were invited to participate, resulting in over 18,000 invitations. Individuals were identified from publicly available information (e.g., institution website) and communication with department chairs by members of the American Institute of Physics Statistical Research Center. Invitations were sent via email, with follow-up reminders sent to those who had not opened the survey at roughly two-week intervals over the course of six weeks.

The initial page of the survey served to inform participants of the nature of the study, their involvement, and potential risk. Informed consent was collected digitally, in accordance with the Institutional Review Board of Western Michigan University policies. The informed consent was followed by eligibility screening questions to ensure that those who participated had been the primary instructor of a general chemistry, single-variable calculus, or introductory quantitative physics course in the 2017–18 and/or 2018–19 academic years that was not taught entirely online. At the end of the data collection period, responses were reviewed and any participants who had not filled out a single post-eligibility question were removed as non-respondents. This resulted in a data set representing 3,769 individuals. Table 1 presents some information about the range of participants included in this study, indicating that the responses are not heavily weighted toward a particular discipline or institution type.

**Table 1:** Table of respondents by institution type, discipline, and rank. Institution type and discipline were ascertained by the research team when the survey roster was developed. Rank was reported by participants and not required, hence the discrepancies in the total; proportion is out of those who provided a response.

*Group*	*Count*	*Proportion*	*Group*	*Count*	*Proportion*
**Institution Type**			**Rank**		
University (UNI)	1541	0.41	Professor	1052	0.33
Predominantly undergraduate institution (PUI)	1129	0.30	Assoc. Professor	692	0.21
Two-year college (TYC)	1099	0.29	Asst. Professor	543	0.17
*Total*	*3769*		Lecturer	773	0.24
**Discipline**			Visiting	102	0.03
Chemistry	1244	0.33	Postdoc	25	0.01
Mathematics	1349	0.36	Grad Student	44	0.01
Physics	1176	0.31	*Total*	*3231*	
*Total*	*3769*		No response	538	*-*

### Data analysis

We conducted analyses to understand to what extent these survey data were consistent with each belief. The beliefs we investigate center on the usage of active learning, which we measure via instructor self-report of how class time is spent [11,17]. In particular, we use the reported percentage of class time spent with students “listening to the instructor lecture or solve problems” (i.e., “in lecture”) as a proxy for the percentage of class time spent in *non-active learning* activities, following others in lumping together various active learning strategies for comparison to traditional didactic lecture [5,6]. After removing responses which did not report on 100% of class time, 3641 survey responses were retained for analysis. Other data used in these analyses (e.g., course enrollment or research activity) also come from self-report survey data, and additional data reduction was performed on a case-by-case basis to omit non-responses; therefore, the N values reported in the findings vary slightly between analyses. Further details of the methods, including data cleaning, data reduction, and tables of statistical results are included in the supplementary materials. For each belief, we present relevant data, analyses, and practical interpretations. In the final section, we summarize the results and suggest implications for policy makers and change agents hoping to increase the use of active learning instruction in introductory STEM courses.

## Findings: Contextual factors

### Course enrollment

**Fig 1:**
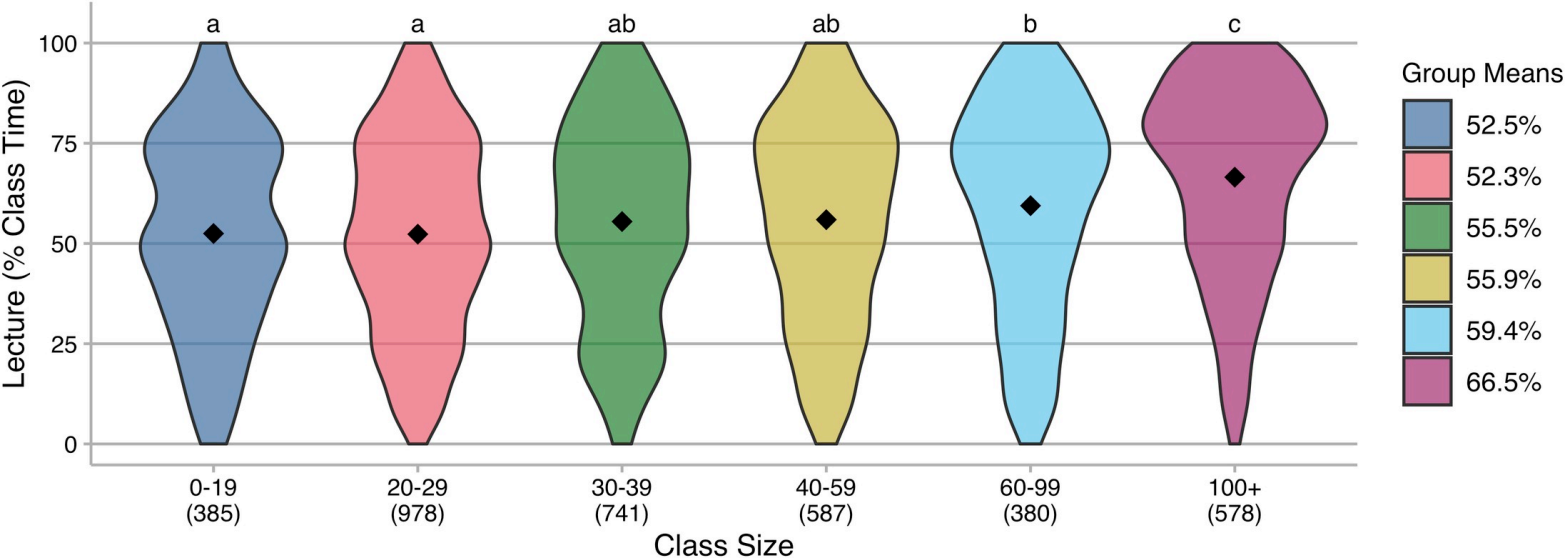
Violin plots of lecture (as percentage of class time) reported by instructors with different class sizes. Group means are indicated in the plots and reported in the legend; N is indicated below each category on the horizontal axis; common letters indicate group means that are not statistically different in Tukey HSD post hoc testing at a 95% confidence level. Class size is a main effect on lecture, *F*(5,3630) = 27.9, *p* < 0.001, η^2^ = 0.04 (small/medium).

One commonly espoused barrier to the use of active learning instructional strategies, particularly those involving student-student engagement, is large class sizes [9,10]. Participants reported the typical enrollment of their course, and these responses were binned into six size categories: 0–19; 20–29; 30–39; 40–59; 60–99; and 100 or more. Responses in the form of a range (e.g., “25–40”) were averaged and binned according to that average (see supplemental information for additional detail). ANOVA indicates a small-to-medium effect [18,19] of class size on percentage of class time spent in lecture (Fig 1). From post hoc testing, the largest classes (those with 100+ students) have the highest percentage of lecture.

The variation we observe in these data suggests that there are instructors in all class size groupings that use active learning, and that there are instructors in all class size groupings that primarily lecture. However, the analysis suggests there is more lecturing in the largest classes. For change agents, this suggests that decreasing class size, particularly avoiding offering very large courses, might result in increased use of active learning in introductory STEM courses. Although our data do not allow us to make causal claims, these results are consistent with other studies indicating that many instructors find large class sizes to be a barrier to the use of active learning [9,10]. Another implication of these findings is that, while lecturing continues to be a norm in introductory STEM, there are a substantial number of instructors who spend very little time lecturing even with large classes. Details of non-lecture activities are included in the supplemental material. These results show that it is possible to use active learning in large classes. We also note that some research-based instructional strategies, such as Peer Instruction [20,21], have been shown to be particularly well-suited to increasing student-student engagement in large courses. Helping instructors select and implement appropriate instructional strategies for their class size, instructional goals, and teaching preferences might be a productive way to increase the use of active learning.

### Classroom setup

**Fig 2:**
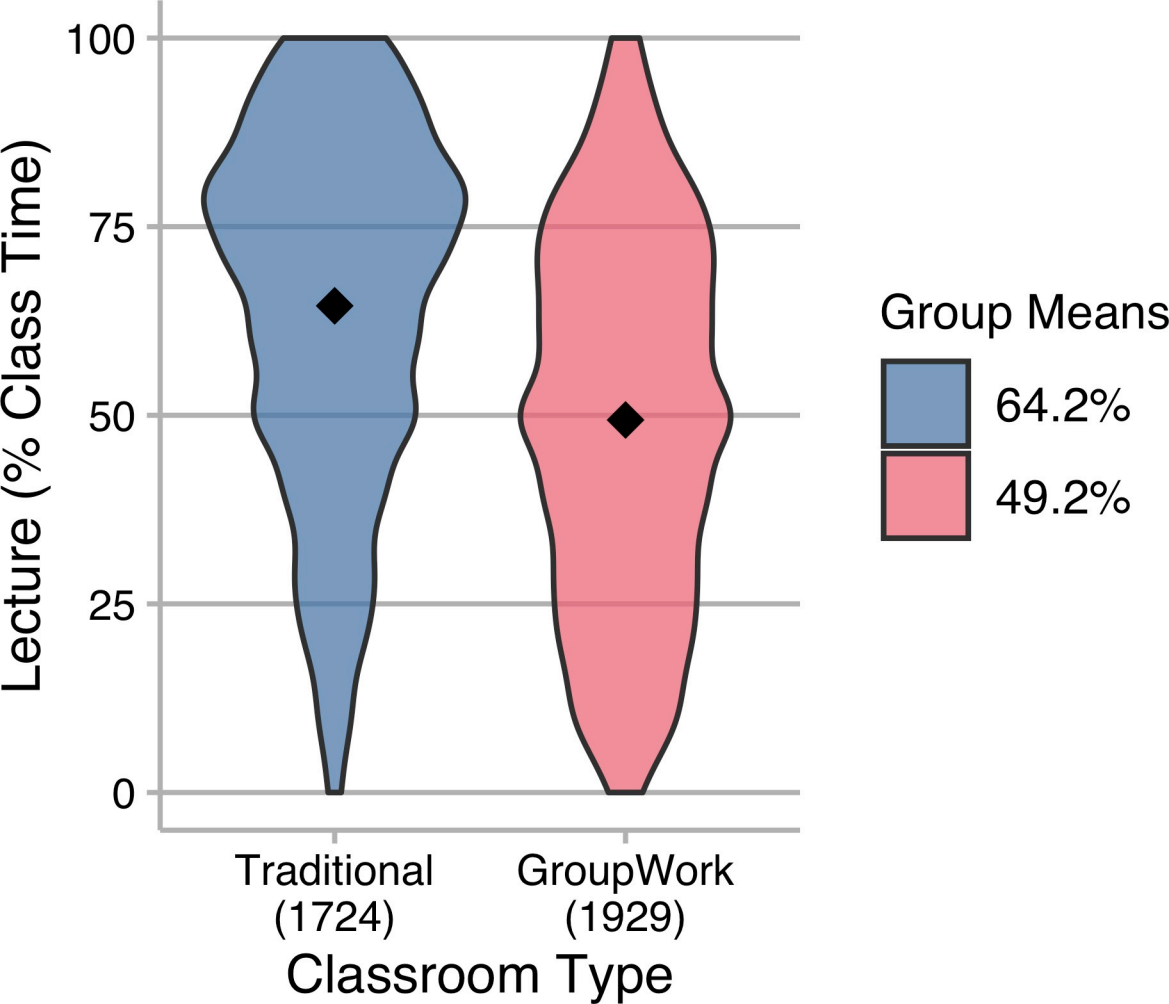
Violin plots of lecture (as percentage of class time) reported by instructors with different classroom types. Group means are indicated in the plots and reported in the legend; N is indicated below each category on the vertical axis; common letters indicate group means that are not statistically different at the 95% confidence level. The amount of lecture used by instructors in different classroom types is different, *t*(3612.2) = 18.98, *p* < 0.001, *g* = 0.63 (medium).

Traditional classrooms are also referenced as a barrier to implementing active learning strategies [10]. Participants in our study indicated whether their classroom was designed to accommodate group work (e.g., tables, movable desks) or were more traditional lecture halls with fixed seats/desks. There is a medium effect [18,19] on the amount of lecture reported by instructors in the two groups, with those teaching in traditional fixed-seat classrooms reporting higher proportions of class time spent in lecture (Fig 2).

As with class size, it is clear that instructors use active learning in both classroom types, and that there are instructors in both types heavily utilizing lecture. However, and consistent with common belief, there is more lecture in traditional fixed-seat classrooms. The medium effect [18,19] size of this result indicates a substantive difference in practice between the two classroom types, on average. Some of this difference may be due to instructors who are more interested in active learning requesting that their classes be assigned to active learning classrooms. For change agents, then, these results suggest that having or building classrooms that physically accommodate peer-to-peer interaction may be an effective way to support active learning instruction, perhaps by making such spaces available to those who already wish to implement active learning strategies. This is consistent with other research that has found multiple benefits for instructors and institutions for creating active learning classrooms [22]. Of course, simply building active learning classrooms does not guarantee that all instructors will use these classrooms as intended. Many institutions that have developed active learning classrooms have reported that instructor training helps to support effective use of these classrooms [22]. Another implication of these data is that there are a substantial number of instructors who lecture very little, even in traditional fixed-seat classrooms. Thus, it is possible to use active learning in these classrooms and more instructors would likely be able to do so with additional support.

### Evaluation of teaching

Student evaluations of teaching (SET) are a common, and often contentious, component of the teaching assessments used in review, tenure, and promotion decisions. Although implementation of SETs can vary from institution to institution, concerns about the use of SETs are widespread, including that they are sensitive to gender biases [23] and are not consistently related to learning outcomes [24]. Additionally, we regularly hear that instructors are less likely to use active learning strategies because they fear that use of such strategies may result in lower SET scores [9,10]. We asked participants two questions about the assessment of teaching: (a) the importance of teaching effectiveness in the overall performance review, and (b) the weighting of SET in the assessment of teaching effectiveness. These five-point Likert-style questions were each collapsed to three levels. Teaching is categorized as a small, medium, or large component of performance review and decisions about promotion; SET are categorized as being given more, equal, or less weight than other measures in the overall assessment of teaching effectiveness (see supplement for additional details).

**Fig 3:**
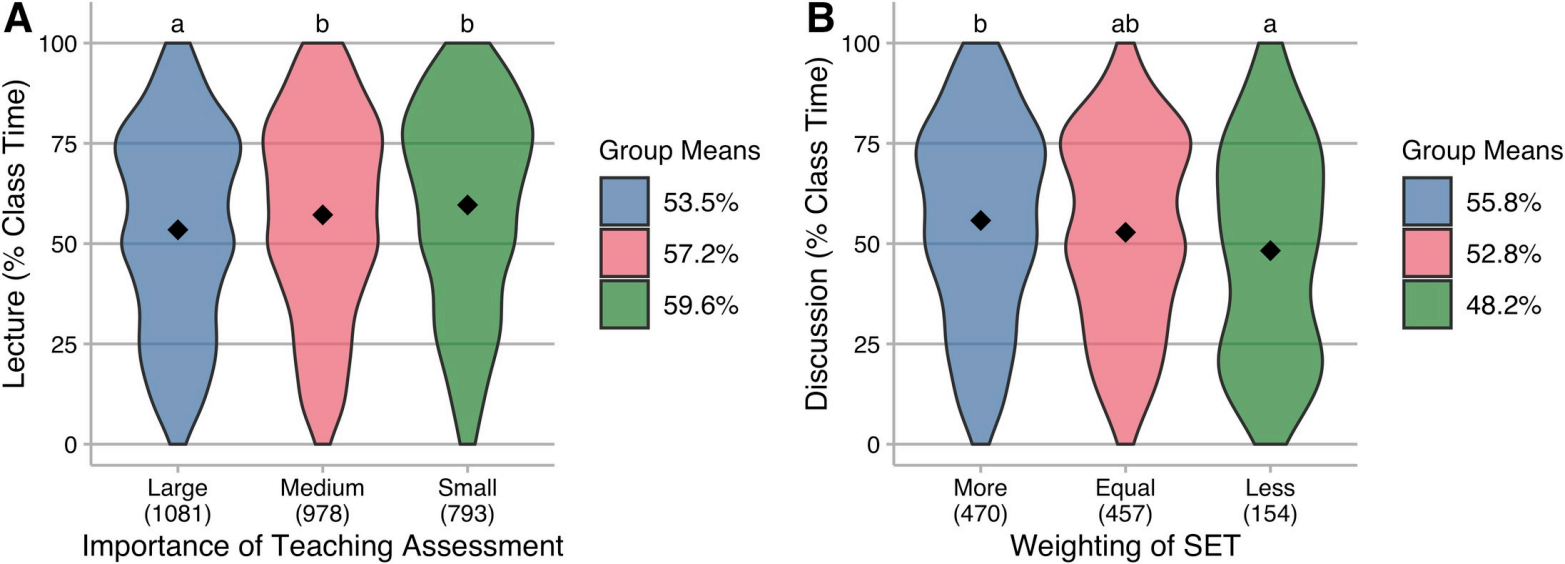
Violin plots of lecture (as percentage of class time) reported by instructors. Group means are indicated in the plots and reported in the legend; N is indicated below each category on the horizontal axis; common letters indicate group means that are not statistically different in Tukey HSD post hoc testing at a 95% confidence level. **A.** All instructors, grouped by the importance of overall teaching assessment for decisions of review and promotion. This is a main effect on lecture, *F*(2,2849) = 14.56, *p* < 0.001, η^2^ = 0.01 (small). **B.** Instructors for whom teaching assessment is of large importance, grouped by the relative weight of SET. This is a main effect on lecture, *F*(2,1078) = 5.63, *p* < 0.01, η^2^ = 0.01 (small).

Among all instructors, an ANOVA test indicates a small effect [18,19] of the importance of teaching evaluation. Instructors who report that teaching assessment is of large importance lecture less than those who report medium or small importance (Fig 3A). To investigate the role of SET, we focused only on those participants who reported that teaching assessment is of large importance. For this subset, ANOVA indicates a small effect of the relative weighting of SET on classroom practice. Those for whom SET is weighted more than other measures (or is the only measure) lecture more than those for whom it is weighted less than other measures (Fig 3B).

Thus, our data are consistent with the belief that reliance on SET as the most important part of teaching evaluation impedes the use of active learning. The variation we observe in these data indicate that instructors can use active learning in varied assessment contexts; it also indicates that there are instructors in all assessment contexts heavily utilizing lecture. However, these data show that instructors report more class time spent in lecture when they believe that teaching assessment is *not* very important in review decisions. Among instructors who report assessment of teaching effectiveness is of large importance in review decisions, lecture increases with increased emphasis on SET (Fig 3B). These results suggest that change agents interested in increasing the use of active learning should work to increase the importance of teaching in performance evaluations and to reduce the importance of SET in the overall evaluation of instruction. This recommendation for reduced emphasis on SET is consistent with recommendations from other research that has found SET are not an appropriate measure of teaching effectiveness and are discriminatory [23,24].

## Findings: Individual factors

### Security of employment

**Fig 4:**
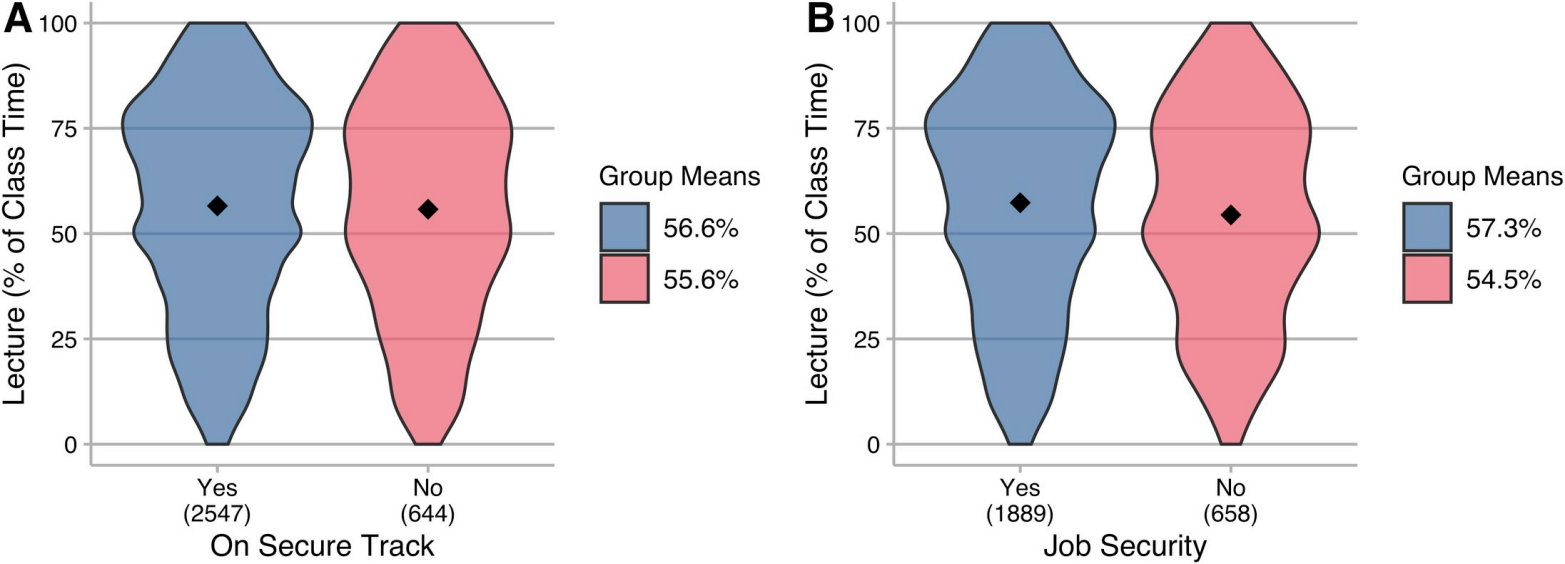
Lecture (as percentage of class time) reported by instructors. Group means are indicated in the plots and reported in the legend; N is indicated below each category on the horizontal axis. **A.** All instructors, grouped by whether or not they are on a track leading to increased job security. There is no evidence of difference between these groups, *t*(984.7) = 0.71, *p* > 0.05, *g* = 0.03 (negligible). **B.** Instructors on a secure track, grouped by whether or not they have achieved increased security. There is no evidence of a difference between these groups, *t*(1185.9) = 2.58, *p* < 0.05, *g* = 0.11 (negligible).

There are many beliefs about instructional practice in relation to academic rank and experience; these are sometimes contradictory. For example, some suggest that job security (e.g., tenure) allows for the flexibility needed to engage in innovative teaching practice, while others argue that new innovations can only be used by instructors from the newest generation who are more innovative and not yet set in their ways. These beliefs drive practice. For example, many change initiatives focus on future faculty or new instructors [25]. Participants reported whether or not they were on a track leading to increased job security, and we saw no difference in the amount of lecture between these two groups (Fig 4A). For those on secure tracks, we have no evidence of a difference in the amount of lecture used by those who have or have not achieved that security (Fig 4B).

Thus, our survey data does not support beliefs about a relationship between security of employment and the use of active learning. The variation and spread of the data suggest that many instructors, regardless of job security or potential for that security, use active learning strategies—and that in all situations many instructors heavily implement lecture. Change agents seeking to increase the use of active learning strategies should not assume that some instructors are more or less likely to be receptive to such practices based solely on their security of employment. That said, individuals in more precarious employment situations may be differentially affected by other factors (e.g., if employment is contingent on teaching assessment), which should be taken into consideration when planning professional development or working to change instructional practice.

### Research activity

Postsecondary instructors are often expected to balance multiple roles. At many institutions, particularly research-intensive universities, the balance between teaching and research activities is often cited as a barrier to instructional change, with many believing that instructors who focus on research are less innovative in their teaching [9]. We asked respondents about the breakdown of their appointment, including the percentage dedicated to research. First we separated instructors with zero and non-zero research appointments, and found no evidence of a difference in the percentage of class time they report spending on lecture (t(2797) = 1.03, p > 0.05, g = 0.04 (negligible).

**Fig 5:**
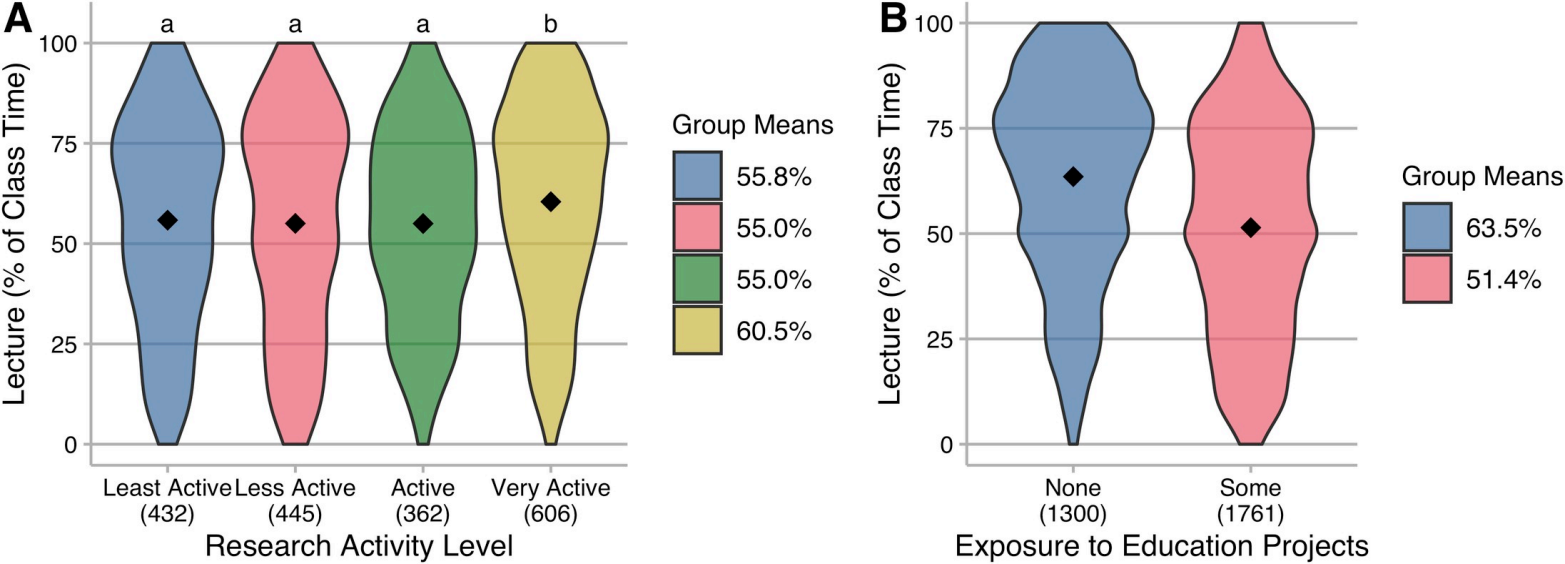
Lecture (as percentage of class time) reported by instructors. Group means are indicated in the plots and reported in the legend; N is indicated below each category on the horizontal axis; common letters indicate group means that are not statistically different in Tukey HSD post hoc testing at a 95% confidence level. **A.** Research activity level is an effect on lecture, *F*(3,1840) = 5.60, *p* < 0.001, η^2^ = 0.009 (small). **B.** Instructors who participate in education research, scholarship of teaching and learning, and/or curricular improvement projects lecture less than those who do not *t*(286.2) = 13.6, *p* < 0.001, *g* = 0.49 (medium).

Among those with a non-zero research appointment, we ranked instructors’ research activity level based on self-report of publications, grants, and presentations (details in supplement). There is a small effect [18,19] of research activity level on the percentage of class time spent in lecture. Post hoc comparison of means shows that very active researchers lecture more than others (Fig 5A). These ‘very active’ researchers reported at least three of the following within the last two years: 20% or greater research appointment; external funding for research; presenting research at two or more professional meetings; submitting two or more manuscripts for publication. Separately, we considered research focus, specifically with an eye toward involvement in education research. Over half of the participants indicated some involvement with education research, which included participating in discipline-based education research, scholarship of teaching and learning, or funded projects aimed at improving undergraduate instruction. There is a medium-sized effect [18,19] of such involvement with education research, and those who *have* been involved use less lecture than those who have not (Fig 5B).

Thus, our data are at least somewhat consistent with the belief that very high levels of research activity may impede the use of active learning. There are two potential implications for change agents interested in promoting the use of active learning. The first is that the majority of instructors who are engaged in research are not engaged to such an extent that it impacts their use of active learning. The second implication is that those instructors with very high levels of research activity do likely have limited time and may need to be supported in implementing active learning strategies that do not increase, or even decrease, time required [26,27]. Perhaps unsurprisingly, instructors who engage in education research or funded education work are more likely to use active learning. Thus, external and institutional grants for instructional development appear to be potentially valuable strategies for improving instruction. For example, instructors with very high levels of traditional research activity may productively participate in instructional development teams [28,29].

### Prior exposure to active learning

**Fig 6:**
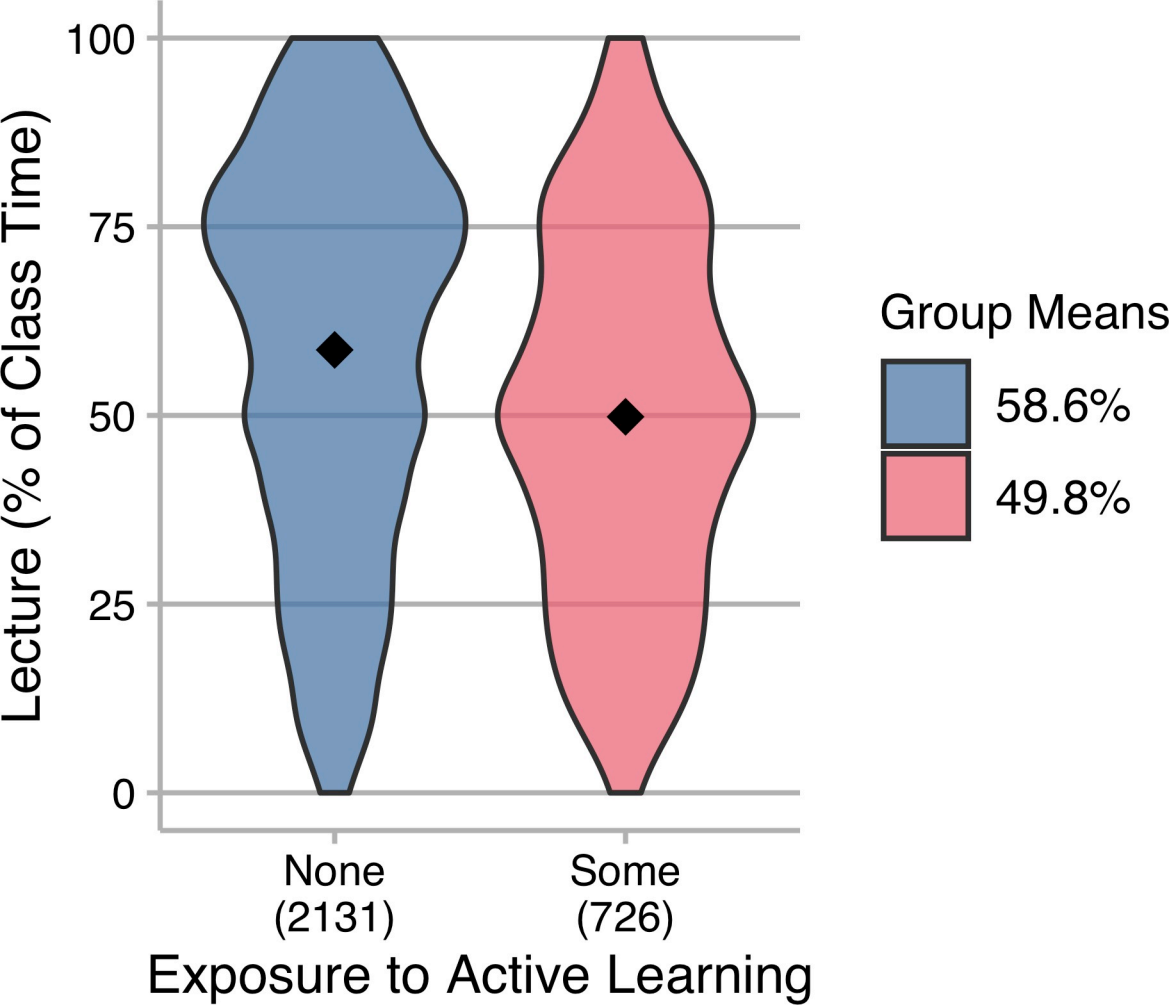
Lecture (as percentage of class time) reported by instructors with different prior experience with active learning. Group means are indicated in the plots and reported in the legend; N is indicated below each category on the horizontal axis. Those who have prior experience with active learning as a student or student instructor lecture less than those who have not *t*(1338.7) = 8.46, *p* < 0.001, *g* = 0.35 (small-medium).

It has been suggested that active learning instructional strategies are not likely to be adopted by instructors until they have personal experience with that instructional style [30], and that experiences as a student form instructors’ beliefs about teaching [31]. Participants in our survey reported on whether they had any experience as a student in an active learning course or as a student instructor (or instructional team member) in a course taught using active learning. We reduced the sample to only those who responded with “Yes” or “No” to each item; consistent with other work on the uptake of innovative instructional practices in STEM, the majority of instructors in our sample (75%) have not been exposed to such instruction as students. Fig 6 shows that instructors who have experienced active learning as a student report lecturing less than those who have not.

The data support the belief that exposure to active learning increases the likelihood that an instructor will use active learning and are consistent with other research about the impact of instructors’ personal experiences [30,31]. For change agents interested in increasing the use of active learning it is, therefore, important to learn about past experiences of instructors and how to build on these. Most of our survey respondents reported having no prior experiences with active learning. It is likely valuable to find ways for them to get such experience, such as co-teaching [32] or participation in a local instructional development team [28,29]. Institutional leaders and change agents should also think about implementation of active learning as not only useful for the current students, but also as the beginning of a cultural change in higher education [9].

## Limitations

In this manuscript we have presented correlational analyses of data from a large survey of chemistry, mathematics, and physics instructors. Our analyses sought to determine whether patterns within these data were consistent or inconsistent with six common beliefs about the implementation of active learning instruction. There are limitations on what we may conclude or imply given the study design and analysis procedures. First, the correlations we report in this paper represent general trends and do not represent the experiences of all individuals. As we discuss, for every variable examined, there are individuals who lecture for most (if not all) of their class time as well as those who lecture sparsely (or not at all). Second, the data we collected does not allow us to make causal claims. We are able to identify patterns (correlations) between different variables, but cannot explain why these patterns exist. Thus, while we do make recommendations in this paper, we do not do so based solely on our data. In all cases recommendations are based on the correlations found in our study as well as on the results of other research. Third, each of the analyses we conduct focus on the correlation between two variables: the amount of lecture reported by instructors vs. another variable, such as self-reported class size. This analysis allows us to accomplish our goal of determining whether the correlations found are consistent with or inconsistent with six common beliefs about the implementation of active learning instruction. However, it is likely the case that there are interactions between the variables tested. For example, instructors who are heavily engaged in research might be more likely to teach large classes. Finally, all data are self-reported. For example, we did not measure the actual amount of class time spent lecturing, but rather relied on self-report from instructors. Although self-report of behavior is not as reliable as observations of behavior, it has been found that instructors are able to self-report general instructional activities, such as percent of class time they spend lecturing, at a reasonable level of accuracy [33].

## Conclusion

We set out to understand the extent to which instructional practice in introductory STEM courses is consistent with six common beliefs about instructor use of active learning instructional strategies. In every context we examined, there are instructors who report using only active learning strategies and instructors who report lecturing for the entirety of class time. While there is much variation, the patterns in survey data collected from 3769 instructors were consistent with the three beliefs about contextual factors, but varied in consistency with the three beliefs about individual factors:

1. Class size. Our data are *consistent* with the belief that large class sizes can hinder the use of active learning. Instructors of very large classes (100 or more students) report significantly more lecturing than instructors of other classes.
2. Traditional classroom. Our data are *consistent* with the belief that traditional fixed-seat classrooms can hinder the use of active learning. Instructors in classrooms designed for active learning use significantly more active learning.
3. Student evaluations. Our data are *consistent* with the belief that emphasizing student evaluations of teaching can hinder the use of active learning. When assessment of teaching effectiveness is important, instructors at institutions with less emphasis on student evaluations report more active learning.
4. Security of employment. Our data are *not consistent* with the belief that not having security of employment (e.g., tenure) can hinder the use of active learning. Instructors without security of employment report using similar levels of active learning to those with security.
5. Research activity. Our data are *somewhat consistent* with the belief that high levels of research activity can hinder the use of active learning. Instructors with very high research productivity report using active learning less than other instructors. On the other hand, instructors who engage in education research or funded curriculum development use more active learning.
6. Experience as a student. Our data are *consistent* with the belief that exposure to active learning as a student or student instructor supports the use of active learning. Instructors who were students in an active learning classroom and/or were part of an instructional team that used active learning are more likely to use active learning instruction.

Those in positions to make structural or policy changes should note these findings and incorporate them into decision-making processes. Institutions trying to increase the use of active learning in undergraduate STEM education should continue to make targeted structural changes such as maintaining and seeking smaller class sizes, building and supporting active learning classrooms, and emphasizing methods beyond SET scores for the evaluation of teaching effectiveness.

These findings also affirm the importance of professional development opportunities, both local and national. As the results show, instructors with all types of individual characteristics and in all types of contexts do manage to incorporate large amounts of active learning into their instruction. Instructors are more likely to implement active learning if they do not have to figure out everything on their own. For example, how to choose active learning strategies that are more compatible with a large class or a particular classroom setup. Professional development activities that focus on local needs and contexts can be highly effective, something which has been incorporated into many current department-based change initiatives [28,29,34].

Our findings reflect national trends in the use of active learning by instructors with different contextual and individual characteristics. There are, of course, many individual instructors who buck these trends and incorporate active learning in their courses regardless of physical setups and cultural norms. How and why they do this needs to be further examined by future research. Similarly, we do not fully understand why some instructors in contexts which seem to support active learning choose to rely on didactic lecture. In summary, the variation we observe in our data suggests that active learning is possible by any instructor in any environment; however, policies can and should be enacted to facilitate and support individual choices to use more active learning in undergraduate STEM courses.

## Supporting information

S1 Table Summary statistics: Percentage of class time spent in lecture by target groups. (DOCX)

S2 Table Results of Welch two sample t-tests: Percentage of class time spent in lecture by target groups. (DOCX)

S3 Table Analysis of variance models: Percentage of class time spent in lecture by target groups. (DOCX)

S4 Table Tukey HSD test at 95% family-wise confidence level: Percentage of class time spent in lecture by target groups. (DOCX)

S1 Dataset (XLSX)

S1 File (DOCX)
