# Supplementary material for: What really impacts the use of active learning in undergraduate STEM education? Results from a national survey of chemistry, mathematics, and physics instructors
Source: PLoS One. 2021 Feb 25;16(2):e0247544. doi: 10.1371/journal.pone.0247544 (PMC7906388; doi:10.1371/journal.pone.0247544)
Supplement: S1 Table — (DOCX) [file pone.0247544.s001.docx]

**Table S1.** Summary statistics: percentage of class time spent in lecture by target groups.

|  | Count | Mean | SD | Median | Minimum | Maximum |
| --- | --- | --- | --- | --- | --- | --- |
| **Binned class enrollment^1^** | | | | | | |
| 0-19 | 380 | 52.49 | 24.34 | 50 | 0 | 100 |
| 20-29 | 973 | 52.32 | 24.12 | 50 | 0 | 100 |
| 30-39 | 740 | 55.47 | 25.26 | 60 | 0 | 100 |
| 40-59 | 587 | 55.94 | 25.36 | 60 | 0 | 100 |
| 60-99 | 378 | 59.43 | 25.86 | 65 | 0 | 100 |
| 100+ | 578 | 66.54 | 24.02 | 70 | 0 | 100 |
| **Classroom setup^1^** | | | | | | |
| Traditional | 1717 | 64.50 | 23.68 | 70 | 0 | 100 |
| Accommodates group work | 1923 | 49.38 | 24.38 | 50 | 0 | 100 |
| **Importance of teaching assessment for review^1^** | | | | | | |
| Large | 1081 | 53.46 | 24.85 | 55 | 0 | 100 |
| Medium | 978 | 57.17 | 25.11 | 60 | 0 | 100 |
| Small | 793 | 59.64 | 25.26 | 65 | 0 | 100 |
| **Weighting of SET compared to other measures of teaching assessment^2^** | | | | | | |
| Heavy / Only | 470 | 55.78 | 24.95 | 60 | 0 | 100 |
| Equal | 457 | 52.82 | 23.96 | 50 | 0 | 100 |
| Light / none | 154 | 48.25 | 26.31 | 50 | 0 | 100 |
| **On secure track^1^** | | | | | | |
| Yes | 2547 | 56.58 | 25.20 | 60 | 0 | 100 |
| No | 642 | 55.79 | 25.35 | 60 | 0 | 100 |
| **With increased security (e.g., tenure)^3^** | | | | | | |
| Yes | 1889 | 57.33 | 25.42 | 60 | 0 | 100 |
| No | 658 | 54.45 | 24.46 | 55 | 0 | 100 |
| **Percentage of appointment dedicated to research^1^** | | | | | | |
| Zero | 1269 | 55.99 | 24.51 | 60 | 0 | 100 |
| Non-zero | 1905 | 56.87 | 25.59 | 60 | 0 | 100 |
| **Research Activity Level^4^** | | | | | | |
| Inactive | 431 | 55.85 | 25.66 | 60 | 0 | 100 |
| Less active | 445 | 55.04 | 27.19 | 60 | 0 | 100 |
| Active | 362 | 55.01 | 23.83 | 57.5 | 0 | 100 |
| Very active | 606 | 60.45 | 25.08 | 60 | 0 | 100 |
| **Involvement with DBER, SoTL, and/or funded education project^1^** | | | | | | |
| None | 1299 | 63.53 | 23.94 | 70 | 0 | 100 |
| Some | 1761 | 51.42 | 24.99 | 50 | 0 | 100 |
| **Experience with active learning as a student and/or student instructor^1^** | | | | | | |
| No | 2127 | 58.68 | 25.71 | 60 | 0 | 100 |
| Yes | 726 | 49.81 | 23.92 | 50 | 0 | 100 |
| 1. All participants who answered the question(s). | | | | | | |
| 2. All participants who answered the question and reported that teaching assessment is very important in decisions of review. | | | | | | |
| 3. All participants who answered the question and reported that they are on a track with the possibility of increased security. | | | | | | |
| 4. All participants who answered the question and reported a non-zero research appointment. | | | | | | |
