# Supplementary material for: What really impacts the use of active learning in undergraduate STEM education? Results from a national survey of chemistry, mathematics, and physics instructors
Source: PLoS One. 2021 Feb 25;16(2):e0247544. doi: 10.1371/journal.pone.0247544 (PMC7906388; doi:10.1371/journal.pone.0247544)
Supplement: S2 Table — (DOCX) [file pone.0247544.s002.docx]

**Table S2.** Results of Welch two sample t-tests: percentage of class time spent in lecture by target groups.

|  | t | df | Sig | Mean diff. | 95% CI of difference | | Hedges’ g | 95% CI of g | | Size of g |
| --- | --- | --- | --- | --- | --- | --- | --- | --- | --- | --- |
|  |  |  |  |  | Lwr. | Uppr. |  | Lwr. | Uppr. |  |
| Classroom setup^1^ | 18.98 | 3612.2 | <0.001 | 15.12 | 13.56 | 16.69 | 0.63 | 0.56 | 0.70 | Medium |
| Secure track^1^ | 0.71 | 984.7 | ns | 0.79 | -1.39 | 2.99 | 0.03 | -0.05 | 0.12 | Trivial |
| Increased security^2^ | 2.58 | 1185.9 | 0.010 | 2.88 | 0.69 | 5.08 | 0.11 | 0.03 | 0.20 | Trivial |
| Research appointment^1^ | 0.96 | 2796.5 | ns | 0.87 | -0.90 | 2.65 | 0.03 | -0.04 | 0.11 | Trivial |
| DBER involvement^1^ | 13.57 | 286.2 | <0.001 | 12.11 | 10.36 | 13.86 | 0.49 | 0.42 | 0.57 | Small-Medium |
| Student experience^1^ | 8.46 | 1338.7 | <0.001 | 8.87 | 6.81 | 10.93 | 0.35 | 0.27 | 0.44 | Small |
| 1. All participants who answered the question(s). | | | | | | | | | | |
| 2. All participants who answered the question and reported that they are on a track with the possibility of increased security. | | | | | | | | | | |
