# Supplementary material for: What really impacts the use of active learning in undergraduate STEM education? Results from a national survey of chemistry, mathematics, and physics instructors
Source: PLoS One. 2021 Feb 25;16(2):e0247544. doi: 10.1371/journal.pone.0247544 (PMC7906388; doi:10.1371/journal.pone.0247544)
Supplement: S3 Table — (DOCX) [file pone.0247544.s003.docx]

**Table S3.** Analysis of variance models: percentage of class time spent in lecture by target groups.

|  | Statistic | Sig. | η^2^ | 95% CI of η^2^ | | Size of η^2^ |
| --- | --- | --- | --- | --- | --- | --- |
|  |  |  |  | Lower | Upper |  |
| Class size^1^ | *F*(5, 3630) = 27.9 | < 0.001 | 0.037 | 0.025 | 0.049 | Small |
| Importance of teaching assessment^1^ | *F*(2, 2849) = 14.56 | < 0.001 | 0.010 | 0.003 | 0.018 | Small |
| Weight of SET in teaching assessment^2^ | *F*(2, 1078) = 5.63 | 0.004 | 0.010 | -0.001 | 0.025 | Small |
| Research activity level^3^ | *F*(3, 1840) = 5.60 | < 0.001 | 0.009 | 0.002 | 0.018 | Small |
| 1. All participants who answered the question(s). | | | | | | |
| 2. All participants who answered the question and reported that teaching assessment is very important in decisions of review. | | | | | | |
| 3. All participants who answered the question and reported a nonzero research appointment. | | | | | | |
