# Supplementary material for: What really impacts the use of active learning in undergraduate STEM education? Results from a national survey of chemistry, mathematics, and physics instructors
Source: PLoS One. 2021 Feb 25;16(2):e0247544. doi: 10.1371/journal.pone.0247544 (PMC7906388; doi:10.1371/journal.pone.0247544)
Supplement: S4 Table — (DOCX) [file pone.0247544.s004.docx]

**Table S4.** Tukey HSD test at 95% family-wise confidence level: percentage of class time spent in lecture by target groups.

| Group 1 | Group 2 | Mean diff. | Lower | Upper | *p* (adjusted) |
| --- | --- | --- | --- | --- | --- |
| **Course enrollment** | | | | | |
| 20-29 | 0-19 | -0.16 | -4.43 | 4.11 | ns |
| 30-39 | 0-19 | 2.99 | -1.47 | 7.44 | ns |
| 40-59 | 0-19 | 3.45 | -1.20 | 8.10 | ns |
| 60-99 | 0-19 | 6.94 | 1.82 | 12.07 | 0.002 |
| 100+ | 0-19 | 14.05 | 9.39 | 18.71 | < 0.001 |
| 30-39 | 20-29 | 3.15 | -0.29 | 6.60 | ns |
| 40-59 | 20-29 | 3.61 | -0.07 | 7.30 | ns |
| 60-99 | 20-29 | 7.11 | 2.83 | 11.39 | < 0.001 |
| 100+ | 20-29 | 14.22 | 10.51 | 17.92 | < 0.001 |
| 40-59 | 30-39 | 0.46 | -3.44 | 4.36 | ns |
| 60-99 | 30-39 | 3.96 | -0.50 | 8.42 | ns |
| 40-59 | 30-39 | 11.07 | 7.15 | 14.98 | < 0.001 |
| 60-99 | 40-59 | 3.49 | -1.16 | 8.15 | ns |
| 100+ | 40-59 | 10.60 | 6.47 | 14.74 | < 0.001 |
| 100+ | 60-99 | 7.11 | 2.44 | 11.78 | < 0.001 |
| **Importance of teaching assessment** | | | | | |
| Medium | Big | 3.72 | 1.13 | 6.31 | 0.002 |
| Small | Big | 6.19 | 3.44 | 8.93 | < 0.001 |
| Small | Medium | 2.47 | -0.34 | 5.28 | ns |
| **Weight of SET in teaching assessment** | | | | | |
| Equal | Heavy | -2.95 | -6.77 | 0.86 | ns |
| Light | Heavy | -7.53 | -12.92 | -2.14 | < 0.001 |
| Light | Equal | -4.58 | -9.99 | 0.83 | ns |
| **Research activity level** | | | | | |
| Less | Least | -0.80 | -5.23 | 3.63 | ns |
| Active | Least | -0.83 | -5.51 | 3.84 | ns |
| Very | Least | 4.61 | 0.47 | 8.74 | 0.022 |
| Active | Less | -0.03 | -4.67 | 4.61 | ns |
| Very | Less | 5.41 | 1.31 | 9.50 | 0.004 |
| Very | Active | 5.44 | 1.08 | 9.80 | 0.007 |
