# Supplementary material for: What really impacts the use of active learning in undergraduate STEM education? Results from a national survey of chemistry, mathematics, and physics instructors
Source: PLoS One. 2021 Feb 25;16(2):e0247544. doi: 10.1371/journal.pone.0247544 (PMC7906388; doi:10.1371/journal.pone.0247544)
Supplement: S1 File — (DOCX) [file pone.0247544.s006.docx]

**Methods details**

**Data cleaning and reduction**

1. During a typical week, what proportion of time during regular class meetings (i.e., lecture sections) do students spend doing the following?
   1. Working individually [Dropdown: 0, 5, 10, …, 95, 100]%
   2. Working in small groups [Dropdown: 0, 5, 10, …, 95, 100]%
   3. Participating in whole class discussion [Dropdown: 0, 5, 10, …, 95, 100]%
   4. Listening to the instructor lecture or solve problems [Dropdown: 0, 5, 10, …, 95, 100]%

Participants were asked to make sure their selections totaled 100. We received 3641 responses which totaled to 100%, 128 which totaled to 0%, and 26 which totaled to something else. The 154 which did not total 100 were removed from the analysis.

1. What is the approximate enrollment in a typical lecture section?
   1. [Text entry]

This resulted in a wide range of entry formats, including ranges (e.g., 20-30) as well as specific values. As we do not expect that people make instructional decisions based on the exact number of students enrolled (e.g., 24 vs. 26 students), we elected to bin enrollments, and to do so based on the average of ranges when those were provided. Three factors were considered in creating these bins: (1) the distribution of responses; (2) our own knowledge of active learning design and usage; and (3) the well-known bins selected by the US News & World report rankings (<https://www.usnews.com/education/best-colleges/articles/ranking-criteria-and-weights>). These bins are: 0-19, 20-29, 30-39, 40-59, 60-99, 100+. In total, 3725 participants answered this item. Four were removed because they answered a different question (e.g., “50 minutes”) and 65 provided ranges, which were then averaged to determine the appropriate bin.

1. Which of the following best describes the set-up in your classroom?
   1. Classroom with fixed seats
   2. Classroom that accommodates group work
   3. Other [Text entry option]

Participants found this question challenging to answer, evidenced by the 228 “other” responses which were submitted along with some form of description or not. These were reviewed by the project team, and a decision was made to adjudicate responses into two groups: (1) physical classrooms that *easily accommodate* group work; (2) physical classrooms that *do not easily accommodate* group work. The original limitation did not account for a range of reported rooms, such as those with fixed rows of tables but with movable chairs.

1. What is the role of student evaluations of teaching (SET) in evaluating teaching performance in decisions of review, promotion, or tenure?
   1. SET are the only measure used to evaluate teaching performance
   2. SET are used and given more weight as compared to other measures
   3. SET are used and given equal weight as compared to other measures
   4. SET are used and given less weight as compared to other measures
   5. SET are not used to evaluate teaching performance
2. How much does the overall assessment of teaching performance matter in decisions of review, promotion, or tenure for someone in your role?
   1. It is not considered
   2. Somewhat influential
   3. Influential
   4. Very influential

Based on the distribution of responses, each of these scales was collapsed to three levels. For #4, (a) and (b) were merged, as were (d) and (e). For #5, (a) and (b) were merged.

1. What is your present academic rank?
   1. Professor
   2. Associate Professor
   3. Assistant Professor
   4. Lecturer/Instructor
   5. Visiting Professor/Lecturer/Instructor
   6. Postdoctoral Instructor
   7. Graduate Student Instructor or Teaching Assistant
2. What is your tenure status at this institution?
   1. Tenured
   2. On tenure track, but not tenured
   3. Not on tenure track, but this institution has a tenure system
   4. No tenure system at this institution
3. Do you have the opportunity for promotion that comes with increased security of employment?
   1. Yes, and I have received such a promotion
   2. Yes, and I have not received such a promotion
   3. No

Those who selected a-d in question #6 were shown question #7; those who selected c-d in question #7 were shown question #8. Classification of employment with the possibility of promotion is associated with 7ab and 8ab; having achieved additional security is associated with 7a and 8a.

1. What is the approximate distribution of your position at [*institution name*]?
   1. Research [Dropdown: 0, 5, 10, …, 95, 100]%
   2. Teaching [Dropdown: 0, 5, 10, …, 95, 100]%
   3. Service [Dropdown: 0, 5, 10, …, 95, 100]%
   4. Administration [Dropdown: 0, 5, 10, …, 95, 100]%
   5. Other [Dropdown: 0, 5, 10, …, 95, 100]%
2. Do you currently have external funding for research?
   1. Yes
   2. No
3. How many professional meetings/conferences have you presented your research or scholarship within the past two years?
   1. [Drop down: 0, 1, 2, 3, 4, 5, 6, 7, 8, 9, 10+]
4. How many articles about your research or scholarship have you submitted for publication within the past two years?
   1. [Drop down: 0, 1, 2, 3, 4, 5, 6, 7, 8, 9, 10+]

For #1, participants were asked to ensure that their responses totaled 100, but of the responses, 554 totaled zero (equivalent to skipping the item) and 49 totaled something else. These were removed, leaving 3166 responses which totaled 100 for analysis.

Median responses for each of these four items were calculated, and respondents received a “research activity point” for each if they were above the median, corresponding to meeting the following criteria:

- Research appointment > 20% (#9)
- External research funding (#10)
- Presenting at two or more professional meetings (#11)
- Submitting two or more articles for publication (#12)

These scores were then converted to activity levels:

- 0 – Least active
- 1 – Less active
- 2 – Active
- 3-4 – Very active

1. Do you conduct STEM education research and/or participate in the scholarship of teaching and learning?
   1. Yes
   2. No
   3. I don’t know
2. In the last five years, have you been part of a project that has received any of the following?
   1. National Science Foundation funding to improve an undergraduate course or the undergraduate curriculum [Yes/No]
   2. External funding (not from the National Science Foundation) to improve an undergraduate course or the undergraduate curriculum [Yes/No]
   3. Internal funding (i.e., from your institution) to improve an undergraduate course or the undergraduate curriculum [Yes/No]

Response to questions #13 and #14 were combined to create an indicator of “involvement with education improvement research.” Those who provided a single “yes” response to any of #13 or #14a-c are considered to have some involvement, while those who provided a “no” to each are not. Responses of “I don’t know” in Question #13 were treated as having skipped the item rather than lumped with either “yes” or “no” responses. Analysis was conducted on the data of the 3061 participants who answered both items.

1. Have you ever been a student in a course taught using RBIS?
   1. Yes
   2. No
   3. I don’t know
2. While a student, have you ever been part of an instructional team for a course taught using RBIS?
   1. Yes
   2. No
   3. I don’t know

Participants who responded with “I don’t know” were removed from the analysis, and a yes to either (or both) questions was considered evidence of prior exposure to active learning while a student.

**Data analysis**

Data reduction, cleaning, combining, and analyses were conducted using RStudio version 1.2.5042 [35 ] and R version 3.6.2 [36 ]. Packages used for data management, analysis, and development of figures: readxl [37 ], reshape2 [38 ], psych [39 ], effsize [40 ], sjstats [41 ], multcomp [42 ], ggplot2 [43 ], ggpubr [44 ], ggthemes [45 ].

When our comparisons involve exactly two groups, we use Welch’s unequal variance *t*-test. In addition to statistical significance, we report standardized effect sizes using Hedge’s *g*. When our comparisons involve factors with more than two levels, we use ANOVA. In addition to reporting *F*-statistics and *p*-values, we report standardized η^2^ estimators of variance. Post-hoc testing is conducted with Tukey’s HSD test, with a family-wise 95% confidence level.

1. RStudio Team. RStudio: Integrated development environment for R. Boston, MA: RStudio, Inc.; 2020. Available: http://www.rstudio.com.
2. R Core Team. R: A language and environment for statistical computing. Vienna, Austria: R Foundation for Statistical Computing; 2019. Available: https://www.R-project.org.
3. Wickham H, Bryan J. readxl: Read Excel Files. 2019. Available: https://CRAN.R-project.org/package=readxl.
4. Wickham H. Reshaping Data with the reshape Package. J Stat Soft. 2007;21. doi:10.18637/jss.v021.i12.
5. Revelle W. psych: Procedures for Psychological, Psychometric, and Personality Research. Evanston, IL: Northwestern University; 2019. Available: https://CRAN.R-project.org/package=psych.
6. Torchiano M. effsize: Efficient Effect Size Computation. Zenodo; 2016. doi:10.5281/ZENODO.1480624.
7. Lüdecke D. sjstats: Statistical Functions For Regression Models. Zenodo; 2018. doi:10.5281/ZENODO.1284472.
8. Hothorn T, Bretz F, Westfall P. Simultaneous Inference in General Parametric Models. Biom J. 2008;50: 346–363. doi:10.1002/bimj.200810425.
9. Wickham H. ggplot2: Elegant graphics for data analysis. Second edition. Cham: Springer; 2016.
10. Kassambara A. ggpubr: “ggplot2” based publication ready plots. 2020. Available: https://CRAN.R-project.org/package=ggpubr.
11. Arnold JB. ggthemes: Extra themes, scales, and geoms for “ggplot2.” 2019. Available: https://CRAN.R-project.org/package=ggthemes.
